# Vicilin—A major storage protein of mungbean exhibits antioxidative potential, antiproliferative effects and ACE inhibitory activity

**DOI:** 10.1371/journal.pone.0191265

**Published:** 2018-02-06

**Authors:** Neha Gupta, Nidhi Srivastava, Sameer S. Bhagyawant

**Affiliations:** 1 School of Studies in Biotechnology, Jiwaji University, Gwalior, India; 2 Department of Bioscience & Biotechnology, Banasthali University, Banasthali, India; Institute of medical research and medicinal plant studies, CAMEROON

## Abstract

Enzymatic hydrolysates of different food proteins demonstrate health benefits. Search for diet related food protein hydrolysates is therefore of interest within the scope of functional foods. Mungbean is one of the popular foods in India because of rich protein source. In this study, mungbean vicilin protein (MBVP) was enzymatically hydrolysed by alcalase and trypsin under optimal conditions. We have studied the antioxidant, antiproliferative and angiotensin-converting enzyme (ACE) inhibitory activities of mungbean vicilin protein hydrolysate (MBVPH) *vis-a-vis* alcalase-generated mungbean vicilin protein hydrolysate (AMBVPH) and trypsin-generated mungbean vicilin protein hydrolysate (TMBVPH). The results showed that MBVPH exhibited higher antioxidant potential, ACE inhibitory and antiproliferative activities than MBVP. The alcalase treated hydrolysate displayed highest ACE inhibitory activity with IC_50_ value of 0.32 mg protein/ml. The MBVP showed significant antiproliferative activity against both MCF-7 and MDA-MB-231 breast cancer cells at the doses between 0.2–1.0 mg/ml. The data suggested that MBVPH can be utilized as physiologically active functional foods with sufficient antihypertensive activity. The results indicate that mungbean can be utilized as a rich resource of functional foods.

## Introduction

Cardiovascular diseases (CVDs) are recognized as major causes of fatalities and presently account for 30% of human mortality [1, 2]. Improving food habits and life style are perceived as the suitable strategies to alleviate CVD. It therefore necessitates the development of nutraceuticals to address these concerns [1]. The nutraceuticals are known to exert several beneficial effects on the human physiology in many ways. Since the synthetic pharmaceuticals have been reported to possess several serious side effects [3], the natural plant based principles are being explored as relatively more effective, safer and cost effective preparations.

The edible vegetative food proteins are major sources of essential amino acids and energy for growth and development of the human body. Food proteins are also important source of biologically active peptides. Recently, the bioactive peptides like angiotensin I-converting enzyme (ACE) inhibitory peptides derived from plant sources have attracted scientific attention towards their roles in protection from CVDs [2].

ACE, a dipeptidyl carboxpeptidase (EC 3.4.15.1), is involved in peripheral hypertension as well as overall cardiovascular functioning. It catalyses the conversion of the inactive decapeptide angiotensin-I into the potent vaso-constricting octapeptide angiotensin-II and also inactivates the potent vasodilator, bradykinin [2]. The inhibition of ACE may result in hypotension. Many potent synthetic ACE inhibitors such as captopril, enalapril, lisinopril and ramipril are widely used in the clinical treatment of hypertension related cardiac failures. These synthetic ACE inhibitors however have been reported to lead adverse side effects such as cough, taste disturbances, rashes and angioedema [3]. Therefore, the quest for searching any natural plant product acting as a chief component of the functional foods for the management of hypertension [4].

A wide range of naturally occurring sources of ACE inhibiton peptides have been extracted from animals, plants and microbial sources. Ovotransferrin [5], shark meat [6] atlantic salmon [7] and hen egg white lysozyme [8] are few such animal sources. Plant derived ACE inhibitory peptides as generated from enzymatic hydrolysates, demonstrate antihypertensive activity both under *in vivo* and *in vitro* systems [4, 9]. Jakubczyk & Baraniak [10] have evaluated ACE inhibitory peptides from digested lentil. Wang et al., [4] have isolated and purified ACE inhibitory peptide of 929 Da molecular weight which had amino acid sequence of Tyr-Val-Pro-His-Trp-Asp-Leu.

Mungbean (*Vigna radiata* (L.) R. Wilczek) is a major pulse crop in India accounting to 16–18 million tonnes production annually. Mungbean seeds contain total protein range of 20 to 30% and are almost free from flatulence-causing factors [11]. The major storage proteins of mungbean are globulin (62%), albumin (16.3%), glutelin (13.3%) and prolamin (0.9%). The vicilin type protein (8S) accounts for 89% of the total globulins containing disulfide linkages and carbohydrates [12].

Enzymatic hydrolysates of different food proteins demonstrate health benefits. Search for diet related food protein hydrolysates is therefore of interest within the scope of functional foods. Earlier reports on mungbean vicilin peptides by Virens et al., [13] have focused mainly on its antihypertensive traits. No further studies so far have been undertaken in this context. The present study is the first report on the mungbean vicilin hydrolysate which has been characterized for its antioxidative potential, antiproliferative properties on the breast malignant cell lines and ACE inhibitory activity.

## Materials and methods

### Materials

1,1-diphenyl-2-picrylhydrazyl (DPPH), 2,2 azinobis (3-ethylbenzo-thiozoline-6-sulfonic acid) disodium salt (ABTS), 6-hydroxy-2,5,7,8-tetra-methylchroman 2-carboxylic acid (Trolox), 2,4,6-tripyridyl-s-triazine (TPTZ), Butylated hydroxyanisole (BHA), N-Hippuryl-His-Leu hydrate (HHL), Alcalase (20 × 10^4^ U g-1, EC 3.4.21.1), Trypsin (25 × 10^4^ U g-1, EC 3.4.21.4) were purchased from Hi Media, Merck and Sigma. Sephadex G-150 was obtained from Sigma Chemical Co. (St. Louis, MO, USA). All other chemicals and reagents used were of analytical grade.

#### Seed material

Mungbean seeds of variety ML-131 (small shiny seeded and partially resistant to yellow mosaic) were obtained from ICAR-National Bureau of Plant Genetic Resources, New Delhi, India following MTA understanding.

Mungbean seed coats were manually removed with scalpel blade. The dehulled seeds were ground into flour using a mortar and pestle and defatted with *n*-hexane (1 g ground seeds: 10 mL hexane) for one hour in an ice bath with constant stirring. The resulting mixture was allowed to stand for 3 min. The solvent was removed by decantation while the residue was air-dried and stored at 4°C until use.

### Methods

#### Extraction and purification of mungbean proteins

The total soluble proteins of mungbean were extracted according to the method of Kortt et al., [14]. Twenty grams of defatted mung bean flour was added with 280 mL of 35 mM potassium phosphate buffer (pH 7.0) containing 0.40 M NaCl. The mixture was stirred for 1 h on ice bath to subjected centrifugation at 12,000 rpm for 5 min at 4°C. The supernatant thus obtained was collected and stored at 4°C until further use. The mungbean protein was obtained and named as MBP. The total soluble protein content of mungbean was determined by the Lowry method with bovine serum albumin as the standard protein [15].

#### Ammonium sulfate fractionation

The crude extract was subjected to ammonium sulphate fractionation. Briefly, to achieve 40% saturation, about 19 gram solid ammonium sulphate was added to 70 ml of crude extract. This mixture was gently stirred for 60 min on ice and then centrifuged at 12,000 rpm for 15 min at 4°C to separate precipitate. The supernatant so obtained was subjected to ammonium sulfate precipitation to achieve 60% saturation. The mixture was further stirred for 60 min and allowed to stand overnight at 4°C. The supernatant was recovered by centrifugation at 10,000 rpm for 10 min.

#### Selective precipitation

The selective precipitation was achieved by dialyzing the supernatant of 60% ammonium sulfate fraction for 48 h against distilled water containing 10 mM β-mercaptoethanol. The dialysate was centrifuged at 12000 rpm for 5 min at 4°C to collect the globulin precipitate. The precipitate was resuspended in minimum amount of extraction buffer and then subjected to gel filtration chromatography and DEAE cellulose chromatography [16].

#### Purification of vicilin

A globulin fraction was loaded onto a gel filtration (Sephadex G-150) column (1x30 cm) to remove other contaminant polypeptides. The sample was eluted with the extraction buffer at a flow rate of 0.4 ml/min. The range of broad eluted fraction showed the presence of vicilin polypeptides on 10% SDS-PAGE. Finally the resulting fraction was purified by DEAE cellulose chromatography. This purified fraction exhibited 4 bands on SDS-PAGE compared with vicilin from other legumes which exhibit more than 3 bands generally. Eluted fractions were subjected for absorbance at 280 nm and analyzed by SDS-PAGE. Fractions showing the band range of 66, 50, 29.5 and 24.4 kDa were pooled and used as a source of vicilin.

#### Preparation of vicilin protein hydrolysate

The vicilin protein hydrolysis was performed according to Yan et al., [17] with slight modifications. The vicilin fraction was mixed with enzyme and hydrolyzed separately with alcalase (20 × 10^4^ U g^-1^, EC 3.4.21.1) at pH 9.5, 60 °C for 2.5 h and trypsin (25 × 10^4^ U g^-1^, EC 3.4.21.4) at pH 8.0, 37 °C for 3.5 h. The pH was kept constant during the entire period of the hydrolysis. After incubation in a water-bath, the enzyme was inactivated at 100 °C for 10 min and the liquid was centrifuged at 12,000 rpm for 10 min in cold. The collected supernatant was lyophilized and named as alcalase-generated mungbean vicilin protein hydrolysate (AMBVPH) and trypsin-generated mungbean vicilin protein hydrolysate (TMBVPH), respectively.

#### Determination of degree of hydrolysis (DH)

The degree of hydrolysis in AMBVPH and TMBVPH was determined according to the method of Hoyle and Merritt [18]. To a 10 ml aliquot of hydrolysate an equal volume of 10% TCA was added. The mixture was incubated for 30 min at 4°C. Thereafter, the mixture was centrifuged at 10,000 X g for 20 min. The obtained TCA soluble protein fraction and the hydrolysate mixture without addition of TCA were each analysed to determine the protein content by method of Lowry [15] using bovine serum albumin (BSA) as a standard. The DH was calculated as the ratio of TCA soluble protein to total protein in the hydrolysate mixture expressed as a percentage.

#### Characterization of proteins

The protein samples were subjected to SDS-PAGE on a Banglore Genei, India mini gel electrophoresis apparatus according to the method of Laemmli [19]. The run was carried out in 11% denaturing gel at 60 V for 4 h. The molecular weights of the subunits were estimated using fermentas protein markers. The gel was stained using 0.05% coomassie brilliant blue R-250 for 2 h then destained and subsequently documented.

#### DPPH radical scavenging assay

Scavenging activity on DPPH free radicals by the extracts was assessed according to the method reported with slight modifications [20, 21]. Briefly, a 2.0 ml solution of the extract, at different concentrations diluted two-fold (2–125 μg/ml) in methanol was mixed with 1.0 ml of 0.3 mM DPPH in methanol. The mixture was shaken vigorously and allowed to stand at room temperature in the dark for 25 min. Blank solutions were prepared with each test sample solution (2.0 ml) and 1.0 ml of methanol while the negative control was 1.0 ml of 0.3 mM DPPH solution plus 2.0 ml of methanol. Thereafter, the absorbance of the assay mixture was measured at 518 nm against each blank with Systronics 2203 UV–vis spectrophotometer. Lower absorbance of the reaction mixture indicated higher radical-scavenging activity. BHA was used as positive control. DPPH radical-scavenging activity was calculated using the equation;

DPPH%=(Ablank-Asample)/Ablank)×100

where A blank is the absorbance of the control reaction (containing all reagents except the sample) and A sample is the absorbance of test sample (with the DPPH solution).

#### ABTS scavenging activity

ABTS (2,2’-azino-bis(3-ethylbenzothiazoline-6-sulphonic acid) discoloration assay was performed with some modifications [22]. Briefly, ABTS^+^ working solution was prepared by reacting 2,2-azo-bis diammonium salt and was diluted to an absorbance of 0.700 ± 0.004 at 734 nm. The reaction mixture consisted of 50 μl sample or standard and 1 ml of ABTS^+^ solution, then vortexed for 30 s. The absorbance was read at 734 nm after 90s of reaction. BHA was used as positive control.

#### Ferric-reducing antioxidant power

The antioxidant capacity of the sample was determined using a modification of the ferric reducing antioxidant power (FRAP) assay [23]. A 1.5 ml sample of the FRAP reagent [10 ml acetate buffer (300 mM, pH 3.6), 1 ml TPTZ (10 mM) in hydrochloric acid solution (40 mM) and 1 ml FeCl3 solution (20 mM)] was added to 50 μl of each sample extract. Absorbance was read at 593 nm immediately after 4 min of incubation at room temperature and the results were expressed as μM Fe^2+^/g of seed extract:

#### Determination of reducing power

Each hydrolysate was used to detect the reducing power using the method of Ahmad et al., [24]. Each 0.2 ml of samples were mixed with 0.75 ml of 0.2 M sodium phosphate buffer (pH 6.6) and 0.75 ml of 1% potassium ferricyanide (w/v). The mixture was incubated for 20 min at 50°C. After incubation, 2 ml of 10% TCA (w/v) was added to the mixture, followed by 10 min of centrifugation at 10,000×g. The upper layer (0.75 ml) was mixed with 0.75 ml of de-ionized water and 0.1 ml of 0.1% ferric chloride (w/v), and the absorbance of the resulting solution was measured at 700 nm. The control was conducted in the same manner, except that distilled water was used instead of sample. Values presented are the mean of triplicate analyses. BHA was used as reference antioxidant.

#### Ferrous ion chelating activity

The method of Decker and Welch [25] was used to investigate the ferrous chelating activity of mungbean protein and hydrolysates. MBVP, AMBVPH and TMBVPH of 0.2 mL were added to 0.2 ml of 2mM FeCl_2_ solution and 0.2 ml of 5mM ferrozine. The mixture was shaken vigorously and incubated at room temperature for 10 min. The absorbance was subsequently measured at 562 nm in the spectrophotometer. The percentage of inhibition of ferrozine—Fe^2+^ complex formation was given below formula:

%chelationactivity=[1-(As/Ac)]x100

where: As—absorbance of sample; Ac—absorbance of control.

#### Antiproliferative effects of mungbean vicilin hydrolysates on human breast cancer cells

The effect of vicilin hydrolysate on breast cancer cell lines i.e. MCF-7 and MDA-MB-231 was studied employing 3-(4,5-dimethylthiazol-2- yl)-2,5-diphenyltetrazolium bromide (MTT) assay [26]. Cells were plated in a 96-well plate at a density of 5,000–7,000 cells per well and grown overnight in 10% fetal bovine serum (FBS). After 24 h, cells were replenished with fresh media and extract was added to the cells. Then 100 μl of different concentrations of the aqueous extracts (10, 25, 50, 75 and 100 mg/ml) of seed sample were added to wells in triplicate. Cells were incubated with the extract for 24 h, after which 20 μl of MTT dye (5mg/ml) was added to each well followed by further incubation for 4 h. Before read-out, formed precipitates of formazon crystal were dissolved in 200 μl of dimethyl sulphoxide (DMSO) using a shaker for 15 min. All steps performed after MTT addition were performed in the dark. Absorbance was measured at 550 nm. The cell % inhibition was calculated using the following formula:

Cellproliferationinhibition(%)=[1-(As/Ac)]x100

where: As—absorbance of sample; Ac—absorbance of control.

#### Extraction of ACE from rabbit lungs

Lungs from rabbit donated by the pharmacology laboratory from Defence Research & Development Establishment (DRDE), Gwalior (Madhya Pradesh) India and were used to obtain the enzyme. Rabbit lungs was extracted according to the method of Cushman and Cheung [27]. Lungs were ground using liquid nitrogen and homogenized in acetone in a ratio of 1 g lung: 2 ml acetone using a homogenizer. The homogenate was centrifuged at 8,000 rpm for 30 min at 4°C and then allowed to stand until the acetone has evaporated. The obtained residue was thus dissolved in 100 mM phosphate buffer, pH 8.3 (1 g residue: 10 ml buffer). The resulting solution was centrifuged at 4,000 rpm and 30 min at 4°C. The clear supernatant was then collected and used as a source of ACE.

#### Spectrophotometric assay for ACE activity

Determination of the ACE inhibitory activities of the digests was done according to the method of Cushman and Cheung [27] with minor modifications. One hundred μl of MBVP, AMBVPH and TMBVPH were added to 500 μl mixture containing 100 μl of 100 mM phosphate buffer (pH 8.3), 100 μl of 300 mM NaCl, 200 μl of 5 mM Hippuryl-L-Histidyl-L-Leucine (HHL) and 100 μl of ACE isolated from rabbit lungs. The mixture was incubated at 37°C for 30 min on incubator shaker and the reaction was stopped by adding 500 μl of 1 N HCl. The reaction mixture was then added with 3.0 ml of ethyl acetate and mixed through vortex for 15 s. Ethyl acetate layer was obtained then the solvent was allowed to evaporate. The residue was redissolved in 1.0 mL of distilled water and the absorbance of the resulting solution at 228 nm was recorded. For the blank, no peptide sample was added. The negative control was devoid of peptide sample. Pulverized captopril served as positive control. ACE inhibitory activities were expressed as inhibitory activity (U), percent inhibition and IC_50_ values. All values are means of three experimental trials.

#### Statistical analysis

All measurements were run in triplicate, and data were expressed as means ± standard deviation (SD). Statistical analysis of the data was done using the Graph pad prism (version 5) software. The differences in mean were calculated using the Duncan multiple-range tests for means with 95% confidence limit (*P <* 0.05).

## Results and discussion

### Extraction and purification of vicilin from mungbean

**Fig 1:**
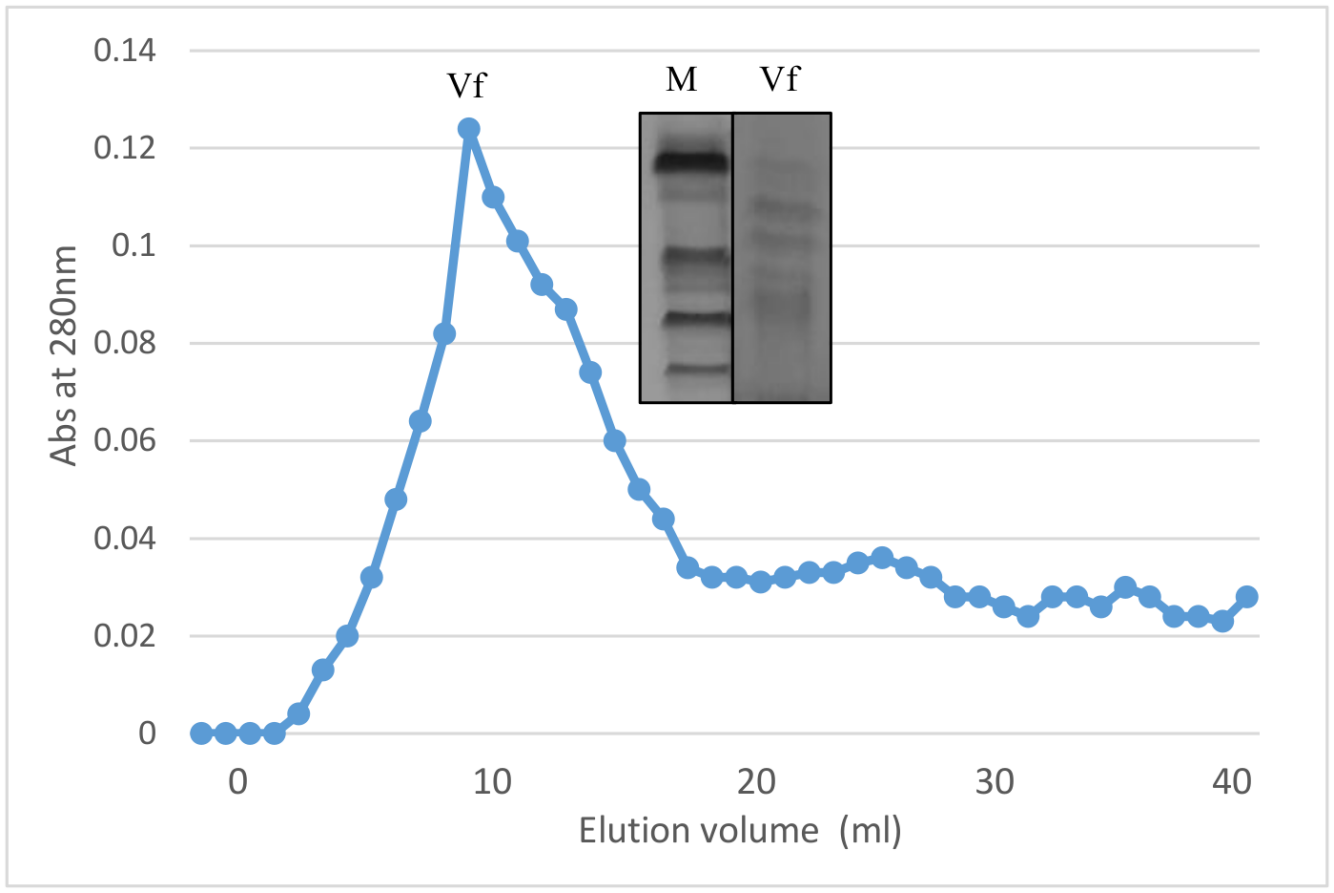
Gel filtration chromatography profile of mungbean vicilin on Sephadex G-150 column. Inset **SDS-PAGE** profile of gel filtration chromatography fractions of vicilin.

**Fig 2:**
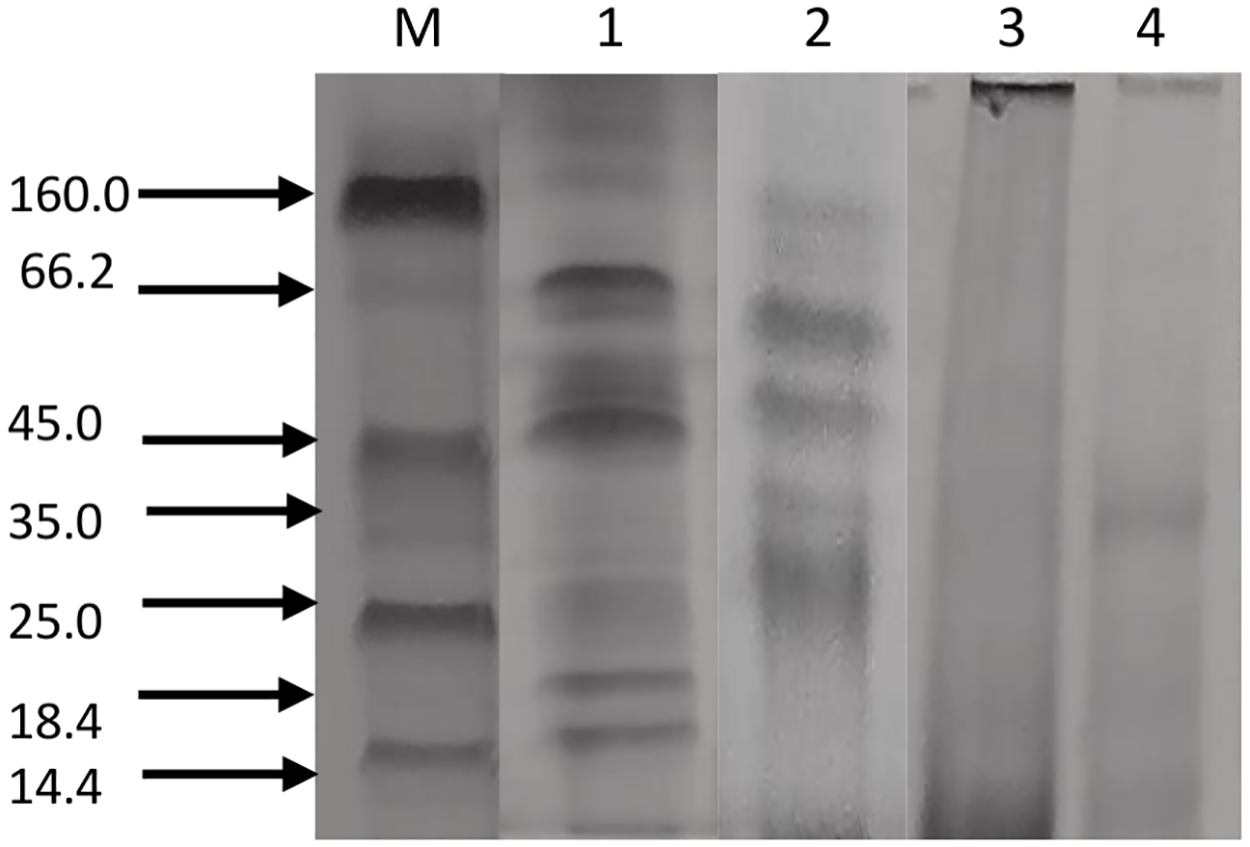
SDS-PAGE profiles of fractions obtained from gel filtration chromatography, Lane M: Molecular weight markers, Lane 1: Total crude extract, 2: Purified vicilin fraction, Enzymatic digestion vicilin Lane 3: alcalase-treated vicilin, Lane 4: trypsin-treated vicilin.

Vicilin is a major seed storage protein of mungbean (*Vigna radiata* L.) and contain bioactive peptides. Vicilin was purified employing ammonium sulfate fractionation and ion exchange chromatography (Fig 1). It was present in the supernatant and further removal of minor storage proteins was achieved using dialysis where globulins were selectively precipitated while albumins remain solubilized. The precipitated globulin fraction subjected to DEAE-cellulose chromatography yielded one major peak, corresponding to the purified vicilin fraction and not corroborated for total soluble proteins of mungbean. Mungbean vicilin consist of four subunits with molecular weights of 66.2, 45.0, 35 and 25 kDa on SDS-PAGE (Fig 2). Similar profile for vicilin fraction was obtained by Ericson and Chrispeels [28] for *Phaseolus aureus*, with molecular weights of 63.5, 50.0, 29.5 and 24.0 kDa. However, mungbean vicilin lacks lower molecular weight subunits like those found in pea [29] and Brazil Nut [30]. Literature perusual suggest that mungbean vicilin peptides has antihypertensive activity. Viernes et al., [13] studied bioactive peptides isolated from mungbean by enzymatic hydrolysis of trypsin and chymotrypsin inhibited ACE activity. They found that chymotrypsin digests yielded peptides were potent than that of trypsin digests.

### Mungbean vicilin protein hydrolysate

**Fig 3:**
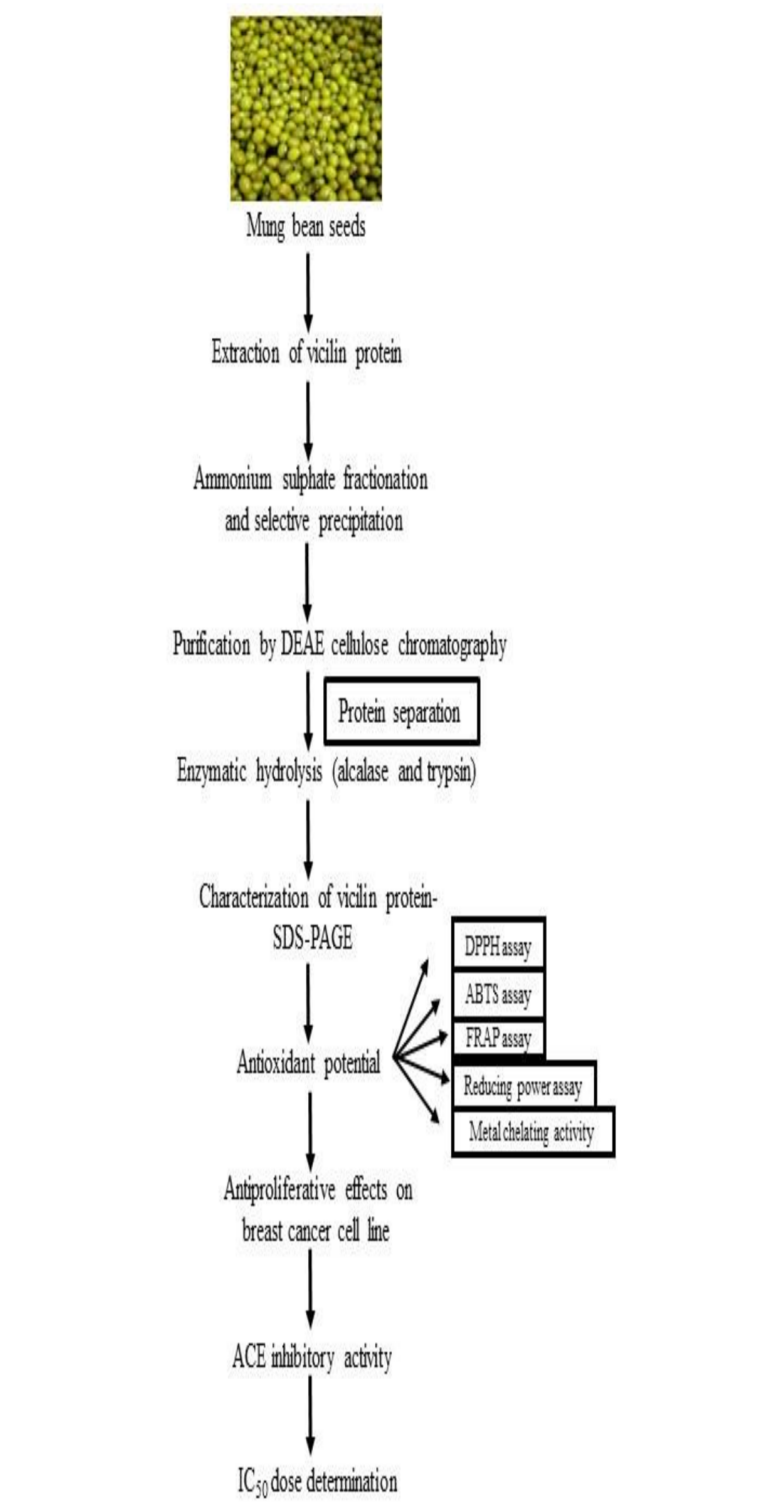
Workflow for vicilin characterization.

The purified vicilin fraction was subjected to enzymatic hydrolysis using alcalase and trypsin. Our study was mainly focused on vicilin, the major storage soluble proteins of mungbean. Therefore, zymography of total soluble proteins of mungbean was deviated. The characterization of vicilin expressed in a workflow (Fig 3). Digestion was fairly complete for both enzymes after reaction as shown by the absence of protein bands on SDS-PAGE (Fig 2). This implies that vicilin has been converted to very small molecular weight peptides that are not resolved and retained by SDS-PAGE. Similar results were observed by Virens et al., [13]. Guang and Phillips [31] reported that diminutive molecular weight peptides with short sequences have the greatest potential to exhibit ACE inhibitory activities since they fit facilely into the active site of the angiotensin-converting enzyme. Wang and Mejia [32] observed that many of these bioactive peptides are of low molecular weight and typically consist of amino acids ranging from 2 to 20 or more. Furthermore, the utilization of long hydrolysis times such as a 24 h period, generally results to the engenderment of ACE inhibitory peptides with more potent activities [33].

**Fig 4:**
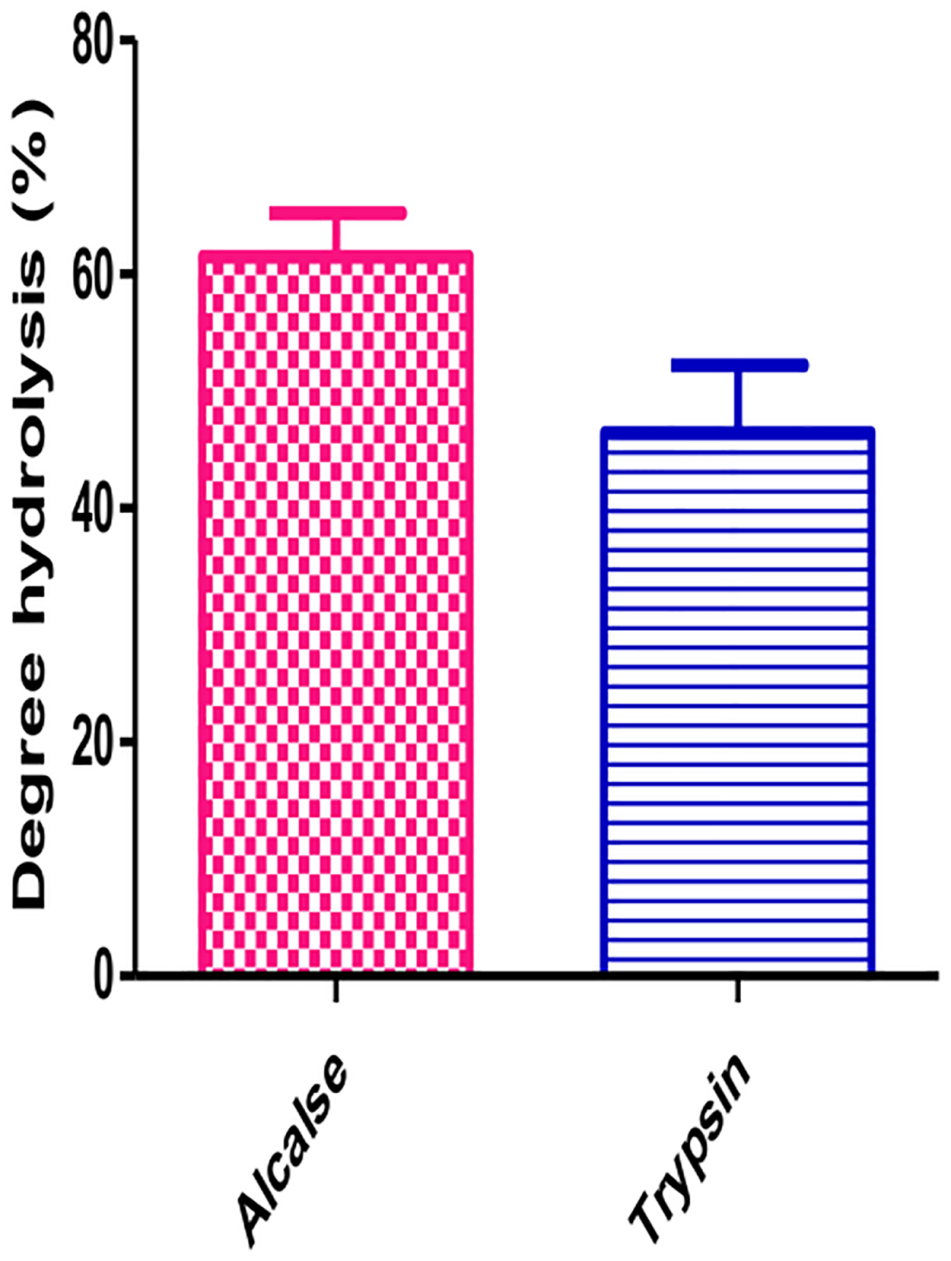
Degree of hydrolysis (DH) of mungbean hydrolysates. Values are means for three measurements.

Alcalase (AL) and trypsin (TH) are microbial preparations, used widely to improve the nutritional and functional properties of food proteins. The main component of alcalase is the serine endopeptidase substilisin [34]. Trypsin catalyzes the hydrolysis of peptide bonds, breaking down proteins into smaller peptides. The ACE-Inhibitory activity of the obtained hydrolysate was measured during 5 h period. The results showed that both hydrolysates demonstrated ACE-inhibitory activity at different levels. In the initial stage (0–5 h), the ACE inhibitory activity hydrolysate was enhanced and reach its maximum after 3.5 hr of hydrolysis. The inhibitory activity of hydrolysates varied from approximately 56.2 ± 2.7 to 44 ± 5.0% and gradually decreased. Therefore, the 3.5 hr incubation was a benchmark for mungbean vicilin hydrolysate. Hydrolysis generating active peptides are crucial and vary from protein to protein. Hamid et al., [35] reported angiotensin-I converting enzyme inhibitory peptide from ostrich egg while and obtained most active hydrolysate after 4 h of hydrolysis. The activities of protein hydrolysates depend on the protein substrate, proteolytic enzyme, time and temperature of hydrolysis as well as amino acid composition and sequence. Mungbean vicilin hydrolyzed with AL and TH showed degree of hydrolysis in a range of 61.5 and 46.4%. The alcalase generated 61.5% degree of hydrolysis in mungbean vicilin while trypsin produced 46.4% (Fig 4). The efficacy of mungbean vicilin as an antioxidant was evaluated using six chemical *in vitro* assays in this study.

The ACE inhibitory activity of mungbean protein was lower than protein hydrolysates. These result shows that proteins have the ability to act as a bioactive compound but because of their large size, they cannot cross cellular membranes. Therefore, hydrolysis is required to generate smaller peptides that possess biological activity. Our results corroborate with the results of earlier workers.

### Free radical scavenging activities on DPPH and ABTS+

**Fig 5:**
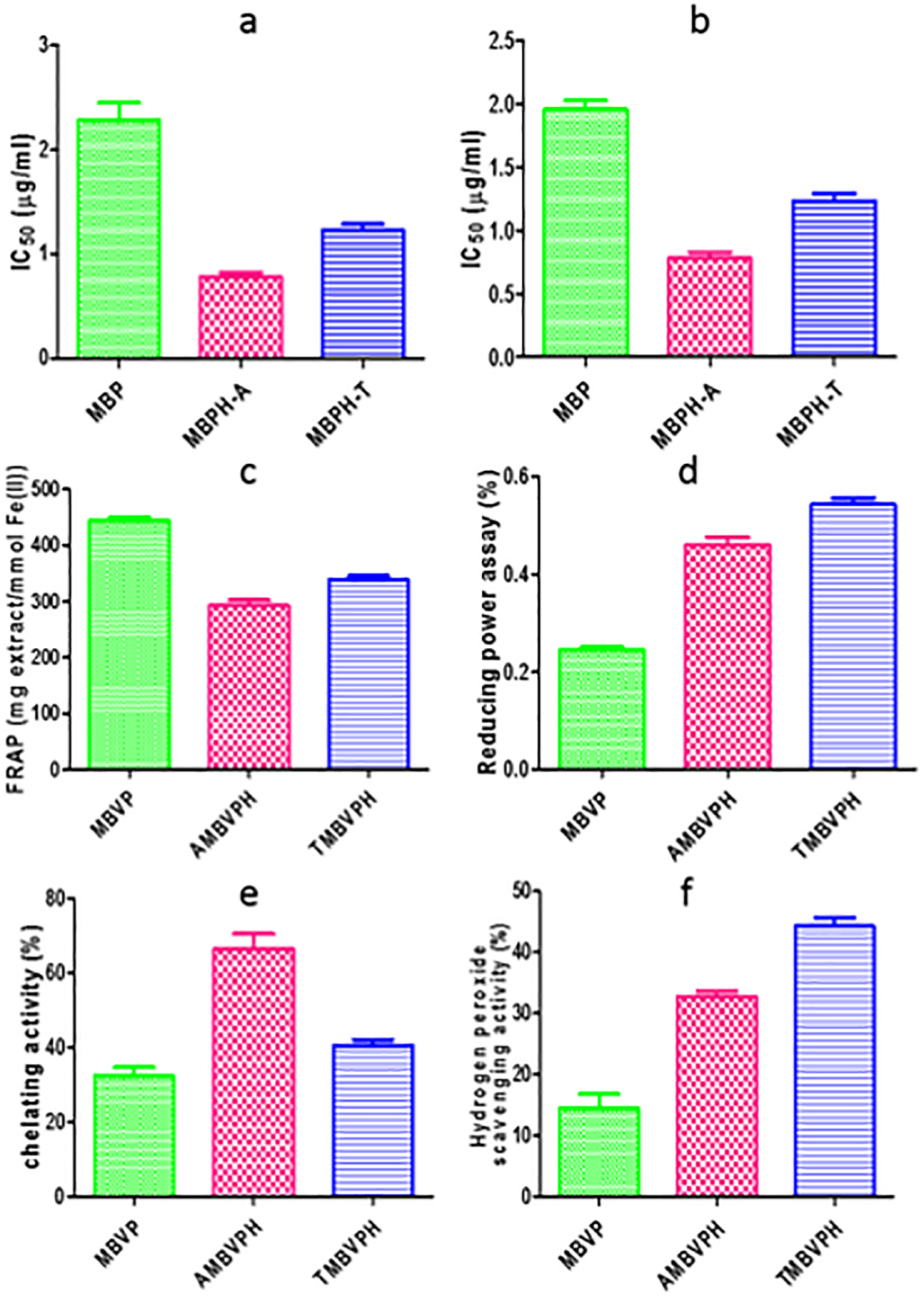
(a) DPPH radical scavenging activity (b) ABTS radical scavenging activity (c) FRAP activity (d) ferrous ion chelating (e) Reducing power (f) Hydrogen peroxide scavenging activity of mungbean seed protein hydrolysates prepared using enzymatic treatment (alcalase and trypsin).

During past decades, a lot of research has been carried out around antioxidants and their effects on health derived from protein hydrolysates of various food sources [36]. DPPH scavenging activity is widely used to evaluate antioxidant activity. Free radicals produced in the body are partly associated with the etiology of cancers and other chronic diseases [37]. Dietary antioxidants, capable of scavenging free radicals, are able to reduce the risk of the diseases. Therefore, it necessitates to determine the radical scavenging effect of antioxidants in pulses. Significant differences (P < 0.05) in DPPH values were found in enzymatic treatments of MBVPH. Among the enzymatic treatments, AMBVPH (IC_50_ at 0.77 μg/ml) had the highest DPPH free radical scavenging activity with lower IC_50_. On the other hand, MBVP (IC_50_ at 1.95 μg/ml) exhibited lowest radical scavenging activity (Fig 5a). This activity was lower than BHA standard. Similar findings were observed in buckwheat where protein hydrolysates showed high DPPH-scavenging activity compared with native buckwheat protein [38].

The ABTS (2,2’-azino-bis(3-ethylbenzothiazoline-6-sulphonic acid) is a colorimetric assay in which the ABTS radical decolorizes in the presence of antioxidants [37]. AMBVPH (IC_50_ at 0.78 μg/ml) exhibited high scavenging ability and low in MBVP (IC_50_ at 2.2 μg/ml). This may be due to generation of phenolics/tannins which has ability to quench free radicals (ABTS+). The higher amounts of phenolics and flavonoids were in correlation with higher antioxidant activity [39].

### Ferric reducing/antioxidant power (FRAP) assay

The FRAP assay was originally applied to plasma, but now commonly used for assay of antioxidants in botanicals [23]. It is characterized by the reduction of Fe ^+2^ to Fe ^+3^ depending on the availability of reducing species followed by the alteration of color from yellow to blue and analyzed through spectrophotometer. The reaction measures reduction of ferric 2,4,6-tripyridyl-s-triazine (TPTZ) to a colored product [40]. The significant differences (P < 0.05) in FRAP values were found among the MBVP, AMBVPH and TMBVPH samples. The ferric reducing ability of the extracts revealed FRAP activity in a range of 292.82 ± 16.40–444.18 ± 11.14 mmol Fe(II)/mg extract (Fig 5). Among the treatments, the highest activity was noted for MBVP (444.52 ± 11.14 mmol Fe (II)/mg extract) and lowest in AMBVPH (292.84 ± 16.40 mmol Fe (II)/mg extract). FRAP assay was used by several authors for the assessment of antioxidant activity of various food product samples [41–44].

### Reducing power

The reducing power was determined using a modified Fe^3+^ to Fe^2+^ reduction assay, whereby the yellow color of the test solution changes to various shades of green and blue, depending on the reducing power of the sample. The presence of antioxidants in the sample causes the reduction of Fe^3+^/ferricyanide complex to the Fe^2+^ form, which is monitored by measuring the formation of Perl’s Prussian blue at 700 nm [24]. The highest reducing power was found TMBVPH, AMBVPH and MBVP (Fig 5) in relation to BHA respectively. Some of synthetic peptides derived from animal egg white protein hydrolysate demonstrating reducing power was earlier reported [45]. Comparing reducing power with other food crops, chickpea protein hydrolysates show much higher activity than wheat [46].

### Metal chelating activity

EDTA, a known metal iron chelator, was used as reference to compare with it the chelating effect of MBVP, AMBVP and TMBVP. The highest activity was noted for AMBVPH (66.28 ± 7.0%) and lowest activity was in MBVP (32.31 ± 3.8%). AMBVPH activity was lower than that of EDTA but higher than that of MBVP. In other pulses like pea, the phaseolin hydrolysates were the most effective iron chelators (81%), having 97% iron chelating activity after treatment with Thermolysin [47]. Gulcin et al., [48] pointed out the chelating agents which forms bonds with metal are effective as secondary antioxidants because they reduce the redox potential, thereby stabilizing the oxidized form of the metal ion. Although the chemical EDTA exhibited the highest (P<0.05) metal chelating activity, natural food derived antioxidants are of growing interest. The incorporation of protein hydrolysate to foods could confer desirable nutritional and functional properties [49]. Ferrous ion chelating effects of vicilin hydrolysates are shown in Fig 5.

### Antiproliferative effects of mungbean vicilin hydrolysates in human breast cancer cells

**Fig 6:**
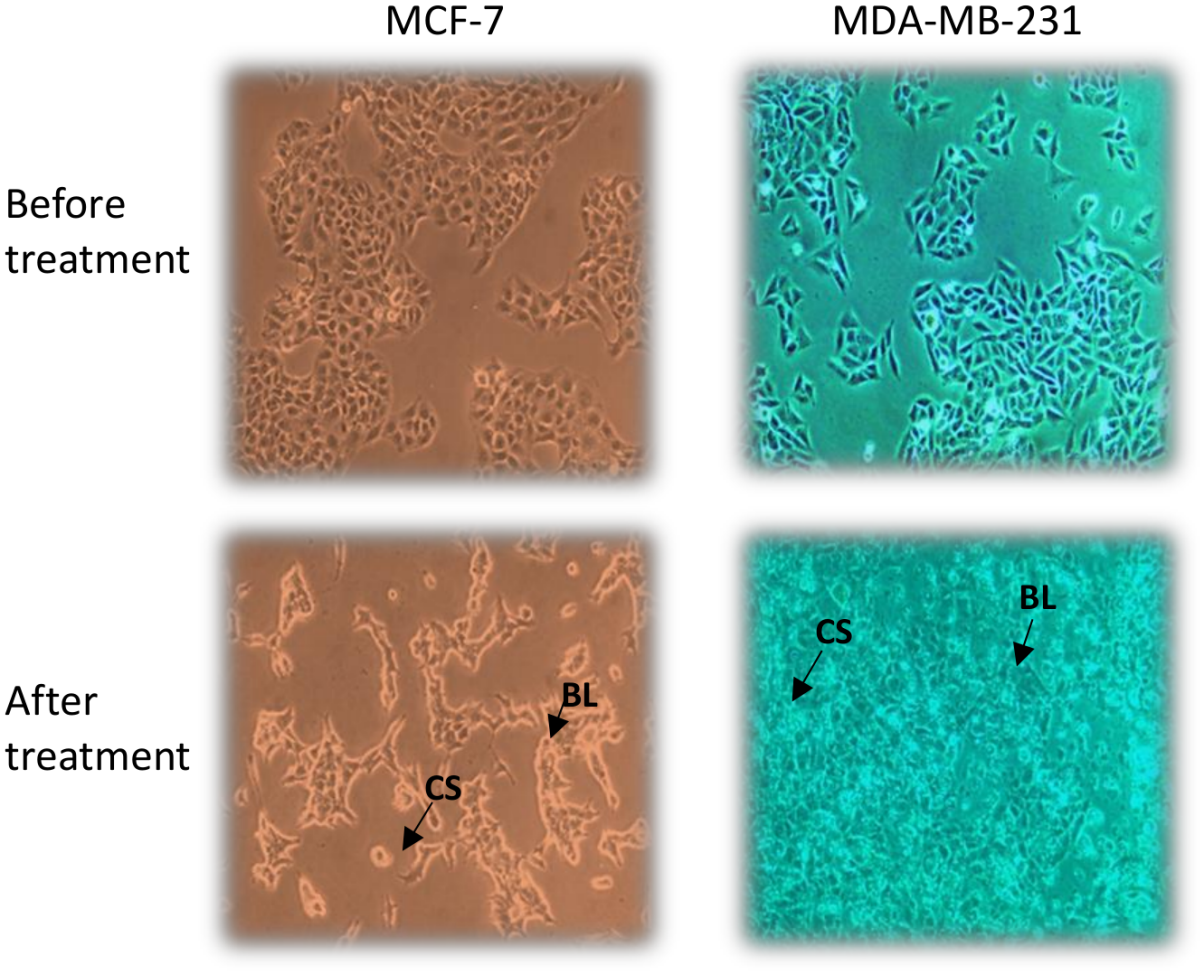
Morphological changes of breast cancer cell line MCF-7 and MDA-MB-231 treated with mungbean protein hydrolysate.

Breast cancer is one of the most common malignancies and heterogeneous cancer among the females worldwide, with an estimated 1.7 million new cases (25.2%) and 0.5 million cancer deaths (14.7%) in 2012 (Cancer, I. A. f. R. o., 2014) [50]. Antiproliferative activity of mungbean seed extracts and their hydrolysate was performed on breast cancer cells viz. MCF-7 and MDA-MB-231 and cytotoxicity was determined (Fig 6). These cell lines differ each other vis-à-vis molecular profile culture doubling times under in vitro conditions and tumorigenicity in vivo. Briefly, MCF-7 cells are molecularly classified as Luminal A (ER+/PR+/HER2-), while MDA-MB-231 is a triple-negative (ER-/PR-/HER2-) and claudin-low cell line. MCF-7 cells are reportedly P53 wild-type, while MDA-MB-231 are P53 mutant. MCF-7 has molecular signature of epithelial phenotype, while MDA-MB-231 are molecularly mesenchymal-like.

**Fig 7:**
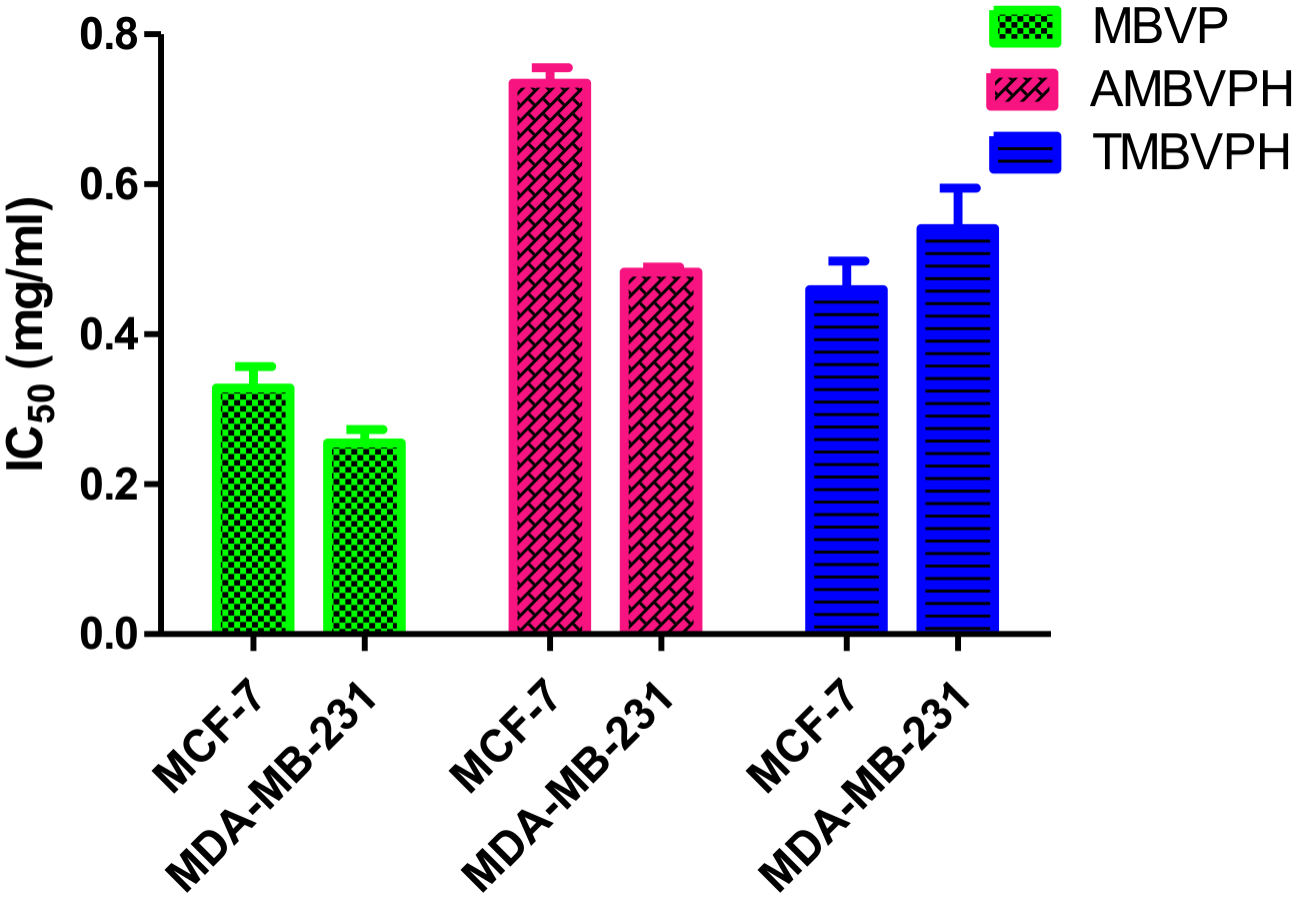
Antiproliferative effect (IC50 -mg/ml) of mungbean seed protein hydrolysates (alcalase and trypsin).

The morphological changes of different concentrations of extracts (0.2mg-1.0mg) showed dose-dependent antiproliferative activities against these cell lines. After 48 h treatment, the IC_50_ of MBVP, AMBVPH and TMBVPH extracts were approximately 0.32 mg/ml, 0.73 and 0.45 mg/ml in MCF-7 cells similarly the IC_50_ of MBVP, AMBVPH and TMBVPH extracts were approximately 0.26 mg/ml, 0.48 mg/ml and 0.54 mg/ml (Table 1). Interestingly only MBVP showed significant dose dependent antiproliferative effect against both cancer cells, with 50% inhibition observed after treating cells with 1mg/ml of MBVP after 48 hr (Fig 7).

**Table 1:** Angiotensin-converting enzyme (ACE) inhibitory activity and antiproliferative effect (as IC50 value) of mungbean protein (MBP), alcalase-generated mungbean protein hydrolysate (AMBPH) and trypsin-generated mungbean protein hydrolysate (TMBPH).

Preparation	ACE inhibitory activity (IC50 mg/ml)	Antiproliferative effect for MCF-7 (IC50 mg/ml)	Antiproliferative effect for MDA-MB-231 (IC50 mg/ml)
MBVP	0.66**	0.32**	0.26**
AMBPH	0.32**	0.73**	0.48**
TMBPH	0.54**	0.45**	0.54**

### ACE inhibitory activity

Hypertension is regulated by the catalytic activity of a zinc protease, called angiotensin-I converting enzyme (ACE) [51]. Thus, ACE plays consequential role in the regulation of blood pressure and hypertension. Commercial ACE inhibitors such as captopril, enalapril, alacepril or lisinopril are often used to treat cardio-related diseases and hypertension [52]. However the inflammatory response, dry cough, taste disturbance or angioneurotic edema are some of the side-effects of such treatments. Most studies on ACE-inhibitory peptides from plant sources have been focused on their production and characterization. Some of the peptides that inhibit the intracellular ET-1 (endothelin-1) also have the potential antihypertensive properties [53]. In the present investigation mungbean vicilin has angiotensin-converting enzyme-I (ACE-I) inhibitory activity and equally have antiproliferative activity. Our reports are in consistency with the reports of Yajun et al., [53].

**Fig 8:**
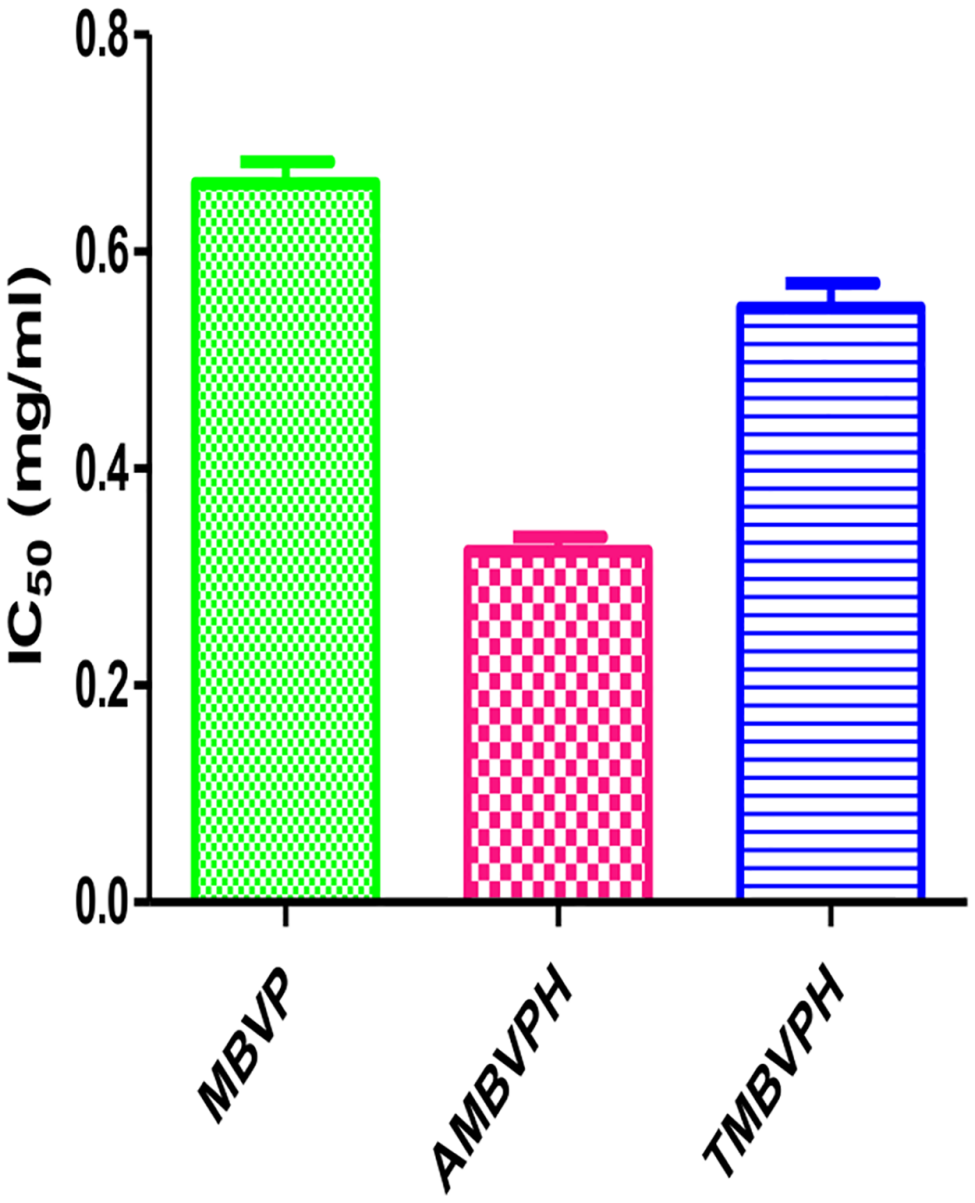
ACE inhibitory activity (IC50—mg/mL) of mungbean seed protein hydrolysates (alcalase and trypsin).

ACE inhibitors from plant protein hydrolysates have gained increasing attention as candidate alternatives to commercially available antihypertensive drugs [54, 55]. Mungbean vicilin hydrolysates in different concentrations (0.2–1.0 mg/mL) were tested for their ACE inhibitory activity and IC_50_ value was determined. The IC_50_ value is defined as the concentration of inhibitor required to inhibit 50% of the ACE inhibitory activity of protein hydrolysates. Among MBVPH, hydrolysates digested with alcalase and trypsin exhibited ACE inhibitory activity an IC_50_ value of 0.66 mg/ml, 0.32 mg/ml and 0.54 mg/ml respectively (Fig 8). The IC_50_ values of MBVPH were much higher than that of captopril, a synthetic ACE-inhibitor drug (0.0000326 mg/mL) [56]. Earlier studies have shown that hydrophobic amino acid residues (leucine, valine, alanine, tryptophan, tyrosine, proline or phenylalanine) preferably bind with catalytic sites of ACE, hence acting as the strong competitive ACE inhibitors [57, 58]. The relationship of KI and IC_50_ for a given compound varies depending on the assay condition and the compound mechanism of inhibition. The relation between KI and IC_50_ is mostly been determined to compare relative potencies of enzyme inhibition compounds [59]. In the present investigation though direct comparison of these values were not accounted mathematically.

Furthermore, the presence of phenylalanine at the third amino acid from the C-terminus of a peptide is reported to be more favorable for the ACE inhibition [57]. Present study demonstrated that MBVPH could be used as an inhibitor to ACE activity while MBVP gave antiproliferative activity against breast cancer cell lines. Aforesaid mungbean seed hydrolysate could be recommended as preventive or/and therapeutic agents mainly for human diseases in addition to prescriptive drugs.

## Conclusion

Alcalase compared to trypsin, was more effective in the hydrolysis of MBVP thus additively suitable for bioactive protein hydrolysates production. The alcalase hydrolysates of MBVP under *in-vitro* conditions showed to be efficient free radical scavengers, having antiproliferative activity against cancer cell line and also ACE inhibitors. The fact that MBVP could be utilized as a source of bioactive hydrolysates may open a new value-addition possibility as an industrial product. Alcalase was shown to be a promising enzyme in further development of bioprocesses for the production of new bioactive food. Further investigations are however necessary to purify and characterize the individual peptides responsible for above *in-vitro* exhibited traits in MBVPH. Thus, MBVPH could finally be recommended as a preventive therapeutic agent for conditions in human diseases in addition or alone as proper prescriptive drug.
